# Impact of Sleeve Gastrectomy on Fecal Microbiota in Individuals with Morbid Obesity

**DOI:** 10.3390/microorganisms11092353

**Published:** 2023-09-20

**Authors:** Danyta I. Tedjo, Jennifer A. Wilbrink, Jos Boekhorst, Harro M. Timmerman, Simon W. Nienhuijs, Arnold Stronkhorst, Paul H. M. Savelkoul, Ad A. M. Masclee, John Penders, Daisy M. A. E. Jonkers

**Affiliations:** 1 Division Gastroenterology-Hepatology, School of Nutrition and Translational Research in Metabolism (NUTRIM), Maastricht University Medical Center+, 6229 Maastricht, The Netherlands; d.i.tedjo@gmail.com (D.I.T.); j.wilbrink@zuyderland.nl (J.A.W.); d.jonkers@maastrichtuniversity.nl (D.M.A.E.J.); 2 Department of Medical Microbiology, School of Nutrition and Translational Research in Metabolism (NUTRIM), Maastricht University Medical Center+, 6229 Maastricht, The Netherlands; paul.savelkoul@mumc.nl (P.H.M.S.); j.penders@mumc.nl (J.P.); 3 Department of Gastroenterology, Zuyderland Ziekenhuis, 6162 Sittard-Geleen, The Netherlands; 4 NIZO Food Research B.V., 6718 Ede, The Netherlands; jos.boekhorst@wur.nl (J.B.); harro.timmerman@wur.nl (H.M.T.); 5 Department of Surgery and Gastroenterology, Catharina Hospital, 5623 Eindhoven, The Netherlands; simon.nienhuijs@catharinaziekenhuis.nl (S.W.N.); astronkhorst@hotmail.com (A.S.); 6 Department of Medical Microbiology & Infection Control, VU University Medical Center, 1081 Amsterdam, The Netherlands

**Keywords:** gut microbiota in obesity, sleeve gastrectomy, intestinal permeability

## Abstract

Background: The intestinal microbiota plays an important role in the etiology of obesity. Sleeve gastrectomy (SG) is a frequently performed and effective therapy for morbid obesity. Objective: To investigate the effect of sleeve gastrectomy on the fecal microbiota of individuals with morbid obesity and to examine whether shifts in microbiota composition are associated with markers of inflammation and intestinal barrier function. Methods: Fecal and blood samples of healthy individuals (n = 27) and morbidly obese individuals pre-SG (n = 24), and at 2 months (n = 13) and 6 months post-SG (n = 9) were collected. The 16SrRNA gene was sequenced to assess microbiota composition. Fecal calprotectin, plasma inflammatory markers and intestinal permeability markers (multi-sugar test) were determined. Results: Fecal microbiota composition between morbidly obese and lean individuals was significantly different. The fecal microbiota composition changed significantly 2 and 6 months post-SG (*p* = 0.008) compared to pre-SG but not towards a more lean profile. The post-SG microbiota profile was characterized by an increase in facultative anaerobic bacteria, characteristic for the upper gastrointestinal tract. No correlations were found between inflammatory markers, intestinal permeability and microbial profile changes. Conclusions: Fecal microbiota composition in morbidly obese individuals changed significantly following SG. This change might be explained by functional changes induced by the SG procedure.

## 1. Introduction

Obesity is an emerging public health problem [1] that is associated with significant co-morbidity [2] and has a substantial impact on direct as well as on indirect healthcare costs [3]. Obesity is associated with a western lifestyle, characterized by an increased intake of energy-dense foods and by a reduction in physical activity. Emerging evidence also points to a role for intestinal microbiota in the etiology of obesity [4,5]. The first evidence was provided by Turnbaugh et al., who showed that the gut microbiota of genetically leptin-deficient obese mice had an increased capacity to extract energy from the diet when compared to that of lean wild-type mice. Moreover, the obese phenotype was found to be transmissible by transplantation of the cecal content of obese donor mice into germ-free lean recipients [6]. In line with these findings, Ridaura et al. [7] showed that the transplantation of fecal microbiota of obese humans to adult germ-free mice resulted in increased total body and fat mass, as well as obesity-associated metabolic phenotypes. In humans, an increased Firmicutes/Bacteroidetes ratio [8,9,10] and reduced microbial diversity have repeatedly been reported in people with obesity as compared to lean individuals [11], but this was not confirmed in all studies [12,13,14]. From a mechanistic point of view, the microbiota may promote weight gain, e.g., by increasing energy harvest via the production of short-chain fatty acids (SCFAs), and by influencing the expression of genes that promote fat storage satiety (e.g., *ANGPTL4*) [15,16,17]. Furthermore, intestinal bacteria can affect the immune system and the expression of tight junctions, leading to intestinal barrier dysfunction and bacterial translocation, thus contributing to inflammation and obesity-related complications (such as non-alcoholic fatty liver disease and type 2 diabetes) [15,17,18,19,20]. As lifestyle modifications are often not successful, surgical interventions are considered a last resort for weight loss and will help to reduce or prevent obesity-related comorbidity [21,22,23]. Both Roux-en-Y gastric bypass (RYGB) and sleeve gastrectomy (SG), as a less invasive bariatric procedure, are frequently applied with good to excellent medium- and long-term outcomes [24,25]. The fecal microbiota changes observed after bariatric surgery are proposed as one of the mechanisms explaining its beneficial clinical outcome [26,27,28]. These changes include an increase in species diversity and changes at the compositional and functional level, but with substantial variability. Whether observed alterations in fecal microbiota are due to weight loss, to altered dietary intake, to consequences of the surgical procedure on gastrointestinal function or to a combination of factors, remains to be established. In general, fecal microbiota changes after sleeve gastrectomy are less pronounced compared to those after RYGB. Only a limited number of studies examined whether SG resulted in a shift in microbial community structure towards the microbiota of lean subjects [29,30,31]. It is relevant to take into account whether an association exists between fecal microbial shifts after sleeve gastrectomy and markers of metabolism, inflammation and of the intestinal barrier [29,30,32,33].

The present study was undertaken to gain further insight (i) into the effect of sleeve gastrectomy on fecal microbiota diversity and composition (ii), and whether shifts in the microbiota are associated with markers of inflammation, intestinal barrier function and glycemic control.

## 2. Material and Methods

### 2.1. Individuals with Morbid Obesity

**Figure 1:**
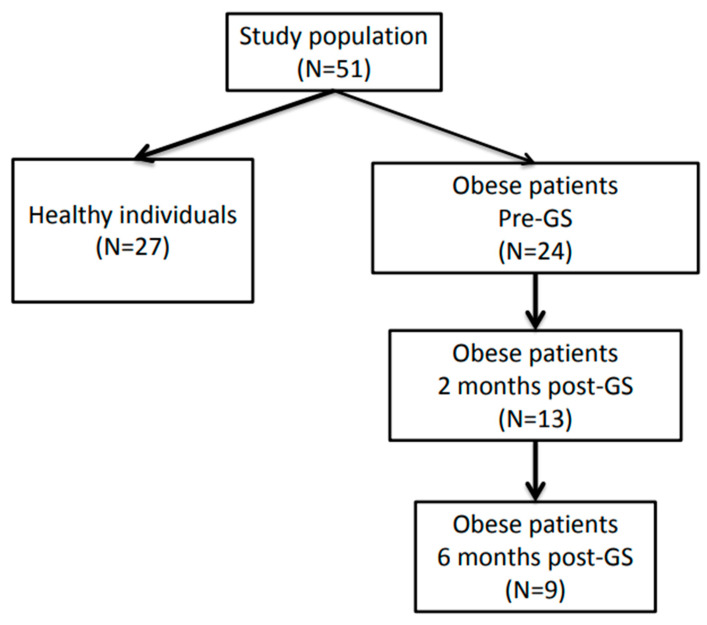
Flowchart of study design.

Individuals with morbid obesity undergoing sleeve gastrectomy at the Catherina hospital Eindhoven, The Netherlands with a stable weight for at least three months prior to surgery, were asked to participate in the current study. In total, 24 morbidly obese individuals between 18 and 65 years of age with either a BMI > 40 kg/m^2^ or a BMI > 35 kg/m^2^ in combination with comorbidities (i.e., diabetes and/or hypertension, dyslipidemia, sleep apnea disorder, GERD and/or osteoarthritis) were included. Exclusion criteria were severe hematological, immunological, neurological, psychiatric, respiratory or cardiovascular diseases; gastrointestinal or hepatic disorders known to influence gastrointestinal absorption, transit and/or microbiota; alcohol consumption >20 units/week; eating disorders; blood donation less than 3 months prior to study participation; pregnancy; lactation; the use of antibiotics less than 4 weeks prior to study participation; and previous gastro-intestinal surgery (except appendectomy and cholecystectomy). The sleeve gastrectomy was performed along a 34 Fr Bougie, starting approximately 6 cm before the pylorus. Patients undergoing SG received a preoperative low-calorie diet (Modifast (500 kcal), Breda, The Netherlands) for two weeks and per-operative antibiotic prophylaxis (single dose of 1000 mg cefazolin). Two months prior to SG (T1), intestinal permeability was determined using a multi-sugar test, and fecal and blood samples for metabolic and inflammatory markers were collected from all 24 patients [34]. A subgroup of patients were willing to repeat the intestinal permeability test and collection of fecal and blood samples two (T2; n = 13) and six months (T3; n = 9) post-SG (Figure 1). All study subjects were requested to refrain from alcohol consumption and to stop proton pump inhibitor (PPI) and NSAID use one week prior to each sampling day. Baseline characteristics and clinical data, including medication use, were collected. Permeability data from this study group had been previously published by our group [35].

### 2.2. Control Subjects

A group of 27 age- and gender-matched healthy lean individuals with a BMI ranging from 18 to 25 kg/m^2^ were recruited by public advertisement, within the context of related studies on fecal microbiota and intestinal permeability, and will further be referred to as lean individuals [36,37]. Exclusion criteria were identical to those for the morbidly obese individuals. Fecal and blood samples were collected using the same, standardized protocol.

### 2.3. Informed Consent Procedure

Written informed consent of each individual was obtained prior to participation. The protocols for the studies of individuals undergoing SG and of the lean controls had been approved by the Medical Ethics Committee of Catharina Hospital Medical Centre (for obese individuals; M11-1109 (CCMO NL34712.060.11)) and the Maastricht University Medical Center+ (for lean individuals; CCMO NL24160.068.08 and CCMO NL31078.068.09) and were executed according to the revised declaration of Helsinki (64th general assembly of WMA, Fortaleza, Brasil, 2013).

### 2.4. Biomarkers in Feces and Blood and Determination of Intestinal Permeability

Fecal samples were frozen at −80 °C within 12 h of defecation until further analysis. To correct for differences in consistency, fecal protein concentrations were determined by the Pierce BCA Protein Assay kit (ThermoFisher scientific, Waltham, MA, USA) as described previously [38]. Fecal calprotectin levels were determined using ELISA (Bühlmann Laboratories, Schönenbuch, Switzerland) according to the manufacturer’s instructions. Blood samples were sent to the laboratory of Clinical Chemistry for routine analysis of high-sensitivity C-reactive protein (hsCRP) and HbA1c in plasma.

The multi-sugar test with 24 h urine collection was performed to assess gastrointestinal permeability in both morbidly obese and lean individuals as described previously [36]. The lactulose-to-rhamnose ratio (L/R) in 0–5 h urine fractions and the sucralose-to-erythritol ratio (S/E) in 5–24 h urine fractions were used as indicators for small- and large-intestinal permeability, respectively. Sucrose excretion in 0–5 h urine was used to determine gastroduodenal permeability.

### 2.5. DNA Isolation of Fecal Samples

A portion of approximately 200 mg was obtained from frozen fecal samples by cutting on ice to prevent the thawing of samples. Subsequently, DNA was isolated using a combination of bead beating and column-based purification with the PSP spin stool kit (Stratec Molecular, Berlin, Germany) as described previously [39]. PCR-grade water was used as a negative control. DNA concentrations were determined using a Quant-IT Pico Green dsDNA reagent kit (Invitrogen, New York, NY, USA) using the Victor3 Multilabel Counter (Perkin Elmer, Waltham, MA, USA).

### 2.6. Sequencing

The V4 region of the 16S rDNA gene was amplified using the 515f/806r primer as described previously [40]. The PCR reaction was performed using 25 µL NEB Phusion High-Fidelity Master Mix (New England Biolabs, Ipswich, MA, USA), 4 µL 515f/806r primer mix and 30 ng metagenomic DNA under the following conditions: denaturation at 98 °C for 3 min, followed by 30 cycles of denaturation at 98 °C for 45 s, annealing at 55 °C for 45 s and extension at 72 °C for 45 s. The final elongation step was at 72 °C for 7 min. Amplicons were purified by using AMPure XP purification (Agencourt Bioscience, Beverly, MA, USA) according to the manufacturer’s instructions, mixed in equimolar concentrations and sequenced on an Illumina MiSeq instrument.

### 2.7. Data Analysis

Participant characteristics are presented as median (with interquartile range (IQR)) for continuous variables and as absolute numbers (proportions) for categorical variables (Table 1). To test for differences between lean healthy and morbidly obese individuals, the nonparametric Mann–Whitney U test and the X^2^ test were used for continuous and categorical variables. The paired nonparametric Wilcoxon signed-rank test and McNemar test were used to test for changes within morbidly obese individuals over time.

Sequences were analyzed using a workflow based on QIIME 1.8 [40]. Operational taxonomic unit (OTU) clustering, taxonomic assignment and reference alignment were performed with the pick_open_reference_otus.py workflow script of QIIME, using Usearch as the clustering method (97% identity) and the GreenGenes database v13.8 as the reference database [41]. Reference-based chimera removal was performed with UCHIIME (Edgar, Haas, Clemente, Quince, & Knight, 2011) [42].

To examine microbial richness and diversity within samples, the following alpha-diversity indices were calculated: Chao1, Shannon index and PD whole tree. The Mann–Whitney U test (MWU) was used to analyze significant differences between lean and morbidly obese individuals, and the Wilcoxon signed-rank test was used to analyze significant changes within obese individuals over time (T1 vs. T2, T1 vs. T3 and T2 vs. T3).

Principal components analysis (PCA) was used to visualize the microbiota structure between the different groups. Subsequently, partial redundancy analysis (RDA) was applied to analyze the microbiota structure between lean and obese individuals, and obese individuals before and after SG. In this partial RDA, we included the variable “subject” as a covariate to remove the variation explained by the repeated measurements. A list of the 20 most important taxa for the separation and their corresponding importance score can be found in Tables S1–S4. To detect potential confounding by diabetes, smoking, medication use, gender and fecal consistency, RDA (for diabetes, smoking, medication use and gender) and Canonical Correspondence Analysis (CCA) (fecal consistency) were performed to examine whether any of these variables were associated with the microbiota composition in lean and/or obese individuals, respectively. CCA and RDA were also used to investigate whether associations were present between the microbiota and markers for inflammation (fecal calprotectin and plasma hsCRP), intestinal barrier function (urinary sucrose, L/R and S/E ratio) and glycemic control (Hba1c). PCA, RDA and CCA were performed in Canoco version 5. The Wilcoxon signed-rank test was used for statistical analysis of differences in the relative abundances of specific taxa between T1, T2 and T3. To correct for multiple testing, the Bonferroni correction was applied. The fold increase or decrease in specific taxa between pre-SG and post-SG was calculated by dividing the relative abundance in post-SG by the relative abundance in pre-SG. The potential relationships between the taxa that were most strongly affected by SG in patients with obesity during follow-up and hsCRP, calprotectin, Hba1c and intestinal permeability markers were investigated using the Pearson product-moment correlation analysis. To correct for multiple testing, the False Discovery Rate method was used. A cut-off value of 0.05 was used.

De-identified data are available upon reasonable requests to be directed to the corresponding author.

## 3. Results

### 3.1. Study Population

Clinical characteristics of lean and morbidly obese individuals are given in Table 1. None of the included individuals used probiotics or antibiotics prior to or during the study, apart from a single dose cefazolin prophylaxis given prior to the SG procedure. Follow-up data were available for 13 and 9 of the obese individuals at two and six months post-surgery, respectively. BMI was significantly higher in surgical patients compared to lean individuals prior to SG (*p* = 9.6 × 10^−10^) and remained significantly higher two (*p* = 8.14 × 10^−7^) and six months (*p* = 8.94 × 10^−6^) after SG. SG resulted in a significant BMI reduction and total weight loss two and six months post-surgery. Hypertension and type 2 diabetes were present in 58.3% and 41.7% of individuals in the surgical group at baseline, respectively, while absent in the controls.

### 3.2. Biomarkers

Two and six months post-SG, HbA1c decreased significantly (*p* = 0.011 and *p* = 0.018, respectively), though in none of the individuals was the resolution of diabetes complete at follow-up. Two morbidly obese individuals (22%) were able to obtain resolution of hypertension following SG.

Inflammation markers hsCRP in plasma and calprotectin in feces were significantly higher in the obese as compared to the lean individuals (*p* < 5.0 × 10^−2^ for all time-points, Table 1). Moreover, hsCRP decreased within the individuals with obesity between baseline and six months post-SG (*p* = 1.1 × 10^−2^). No decrease was found for fecal calprotectin post-SG.

Urinary sucrose excretion (*p* = 6.0 × 10^−3^), but not the L/R and S/E ratios, was significantly higher in the obese individuals (T1) versus lean individuals. Post-SG, L/R ratio decreased significantly after six months (T1 vs. T3, *p* = 8.0 × 10^−3^) and the S/E ratio decreased significantly after two months (T2) when compared to baseline (T1) within the operative group (*p* = 1.6 × 10^−2^). Fecal protein content did not significantly differ between groups.

### 3.3. Sequencing Data

A total of 3,267,977 raw sequences were obtained from 73 samples (n = 24 healthy controls HC; n = 24 morbidly obese T1, n = 13 T2 and n = 9 T3). After quality filtering and binning, 2,405,726 sequences with a median of 29,867 sequences per sample (IQR: 26,611 35,723) were available for downstream analysis.

### 3.4. Richness and Diversity of Fecal Microbiota of Obese and Lean Individuals

**Figure 2:**
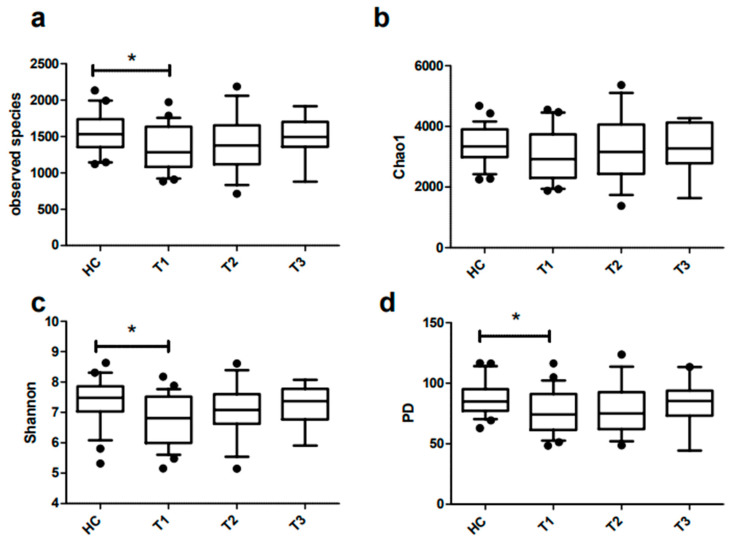
Alpha diversity indices (observed species (**a**), Chao1 (**b**), Shannon diversity index (**c**) and PD whole tree (**d**)) of healthy individuals (HC) and patients with obesity pre (T1), two (T2) and six months (T3) post-SG. Data are presented as boxplots displaying medians with interquartile ranges. * indicates *p* < 0.05.

When compared to the lean individuals, the microbiota of morbidly obese individuals was characterized by a significantly lower microbial richness, as indicated by the number of observed species (median [IQR]: 1529.6 [IQR: 1351.6–1735.6] vs. 1280.3 [1079.4–1631.9]; *p* = 0.02). The richness as estimated by the Chao1 index was also lower, but the difference was not statistically significant. Moreover, the microbial diversity, taking into account both microbial richness and evenness, was also significantly lower in obese individuals when assessed by the Shannon diversity index (6.8 [6.0–7.5] vs. 7.5 [7.0–7.9]; *p* = 0.02) as well as by the phylogenetic diversity metric PD whole tree (74.1 [61.4–91.0] vs. 85.0 [77.2–95.0]; *p* = 0.02) Figure 2). Two and six months post-SG, the microbial richness and diversity of obese individuals was still lower as compared to lean individuals, but this difference was no longer significant.

### 3.5. Fecal Microbiota at the Phylum Level

**Figure 3:**
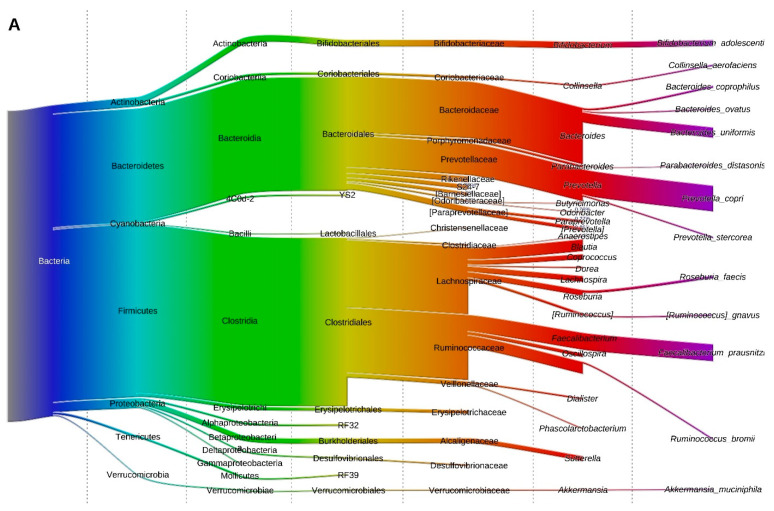

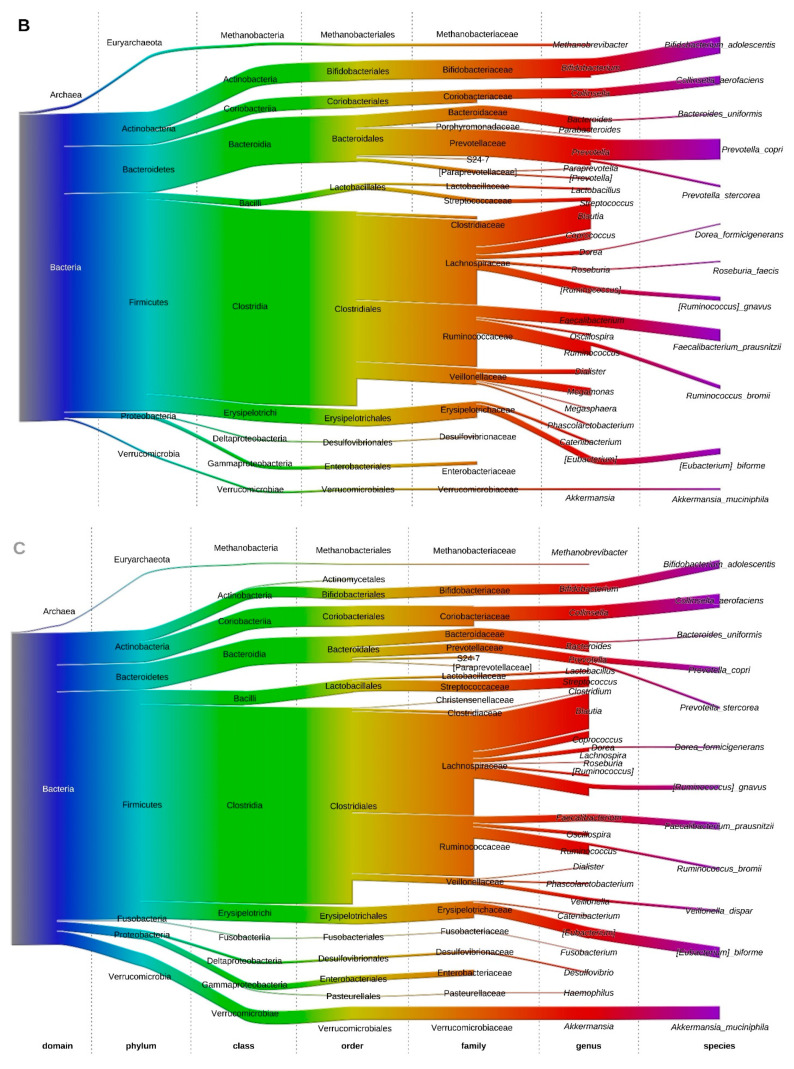

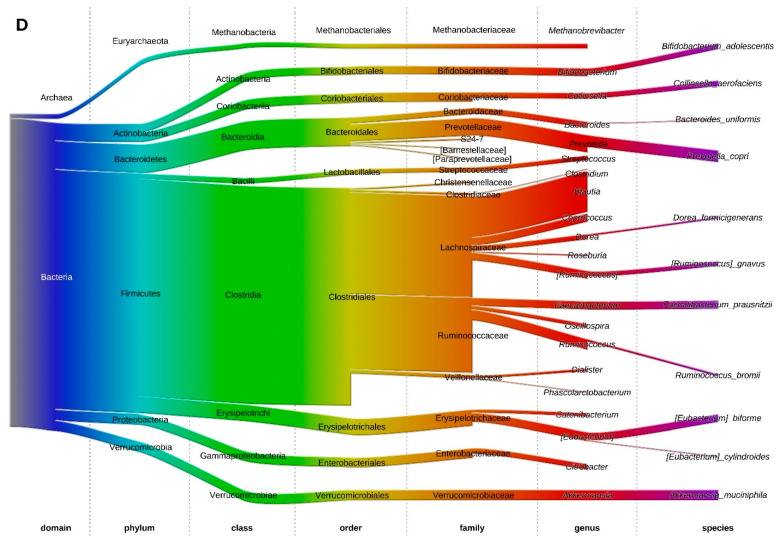
Visual representation of the microbiota composition in healthy, lean individuals (**A**) and patients pre-SG (**B**), two (**C**) and six months (**D**) post-SG. The microbiota is presented from the domain to the species level.

The microbiota composition at the phylum level of lean individuals and obese individuals pre- and post-SG is shown in Figure 3. Before surgery, the obese individuals had a significantly lower relative abundance of Bacteroidetes (15.1% vs. 36.4%, resp., *p* = 9.5 × 10^−7^), Proteobacteria (1.8% vs. 3.2%, resp., *p* = 2.1 × 10^−4^) and Tenericutes (1.3 × 10^−3^% vs. 7.6 × 10^−3^%, resp., *p* = 91.0 × 10^−3^) as compared to lean individuals. Firmicutes (70.5% vs. 55.5% resp., *p* = 1.2 × 10^−4^) and Actinobacteria (0.1% vs. 0.03%, resp., *p* = 1.2 × 10^−7^) were significantly more abundant in prior to SG as compared to lean individuals. In line with this observation, the Firmicutes/Bacteroidetes (F/B) ratio was significantly higher in the obese as compared to lean individuals (*p* = 3.00 × 10^−5^, Figure S1).

### 3.6. Fecal Microbiota Structure of Obese and Lean Individuals

**Figure 4:**
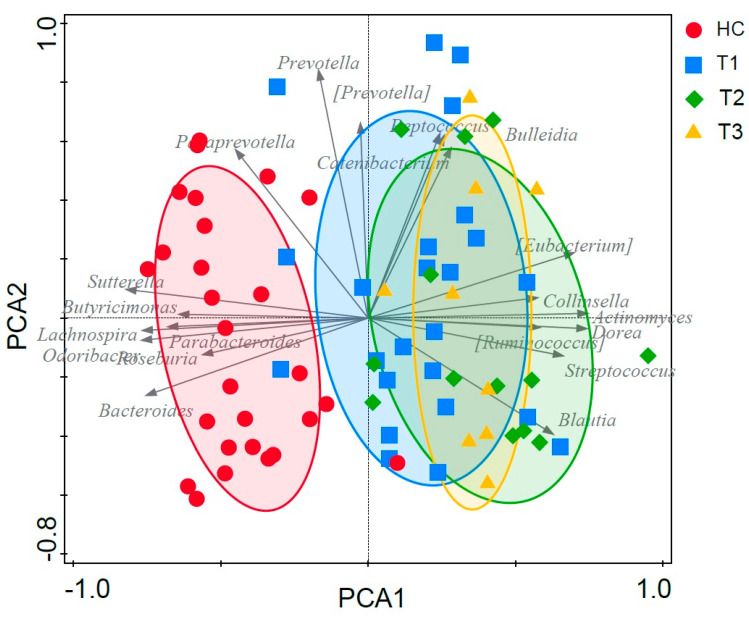
PCA based on the fecal microbiota composition of healthy individuals (red circle), individuals with obesity pre (T1; blue squares), two (T2; green diamonds) and six months (T3; orange crosses) post-SG. The ellipses are 2D-normal-based ellipses at the 66% confidence level. Horizontal axis: “PCA1”, vertical axis: “PCA2”.

The clustering of fecal samples by means of PCA indicated that the overall fecal microbial community structure of individuals with morbid obesity at baseline (pre-SG) could be clearly separated from that of lean individuals. Furthermore, the fecal microbiota composition of obese patients two and six months post-SG tended to cluster apart from the microbiota of obese individuals pre-SG, but did not change towards that of lean individuals (Figure 4).

**Figure 5:**
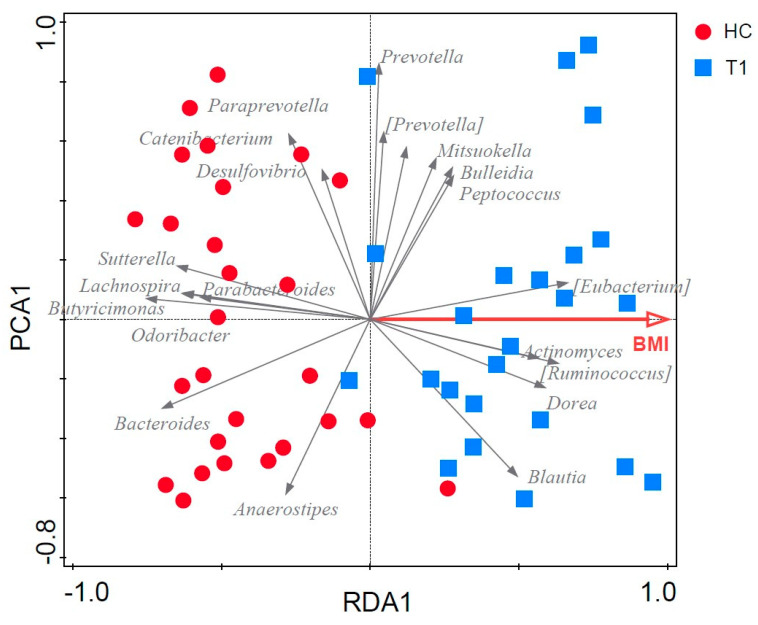
RDA based on the fecal microbiota composition of healthy individuals (circles) and patients pre-GS (squares). The 20 taxa that contributed most to the separation are represented by arrows. The effect of the variable BMI is represented by the red arrow. Horizontal axis: “RDA1”, vertical axis:”PRCA1”.

To further investigate the baseline differences in microbiota composition of obese and lean individuals, a constrained ordination method, RDA, was performed. RDA confirmed the separation of fecal samples by health status and showed that BMI significantly contributed to explain the variation in microbiota composition (variance explained = 12.2%, Monte Carlo permutation test: *p* = 0.002) (Figure 5). This separation was driven by a higher relative abundance of the genera *Odoribacter* (*p* = 7.35 × 10^−5^)*, Bacteroides* (*p* = 4.11 × 10^−4^), *Sutterella* (*p* = 2.14 × 10^−5^)*, Lachnospira* (*p* = 2.04 × 10^−5^), *Butyricimonas* (*p* = 2.02 × 10^−5^) and *Parabacteroides* (*p* = 0.04) in lean individuals and a concomitant higher relative abundance of *Eubacterium* (*p* = 0.02)*, Dorea* (*p* = 5.13 × 10^−3^) and *Ruminoccoccus* (*p* = 2.56 × 10^−3^) in obese individuals (Figure 5). A list of the 20 most important taxa for the separation and their corresponding importance score can be found in Supplemental Table S1. Including “BMI” as a covariate to remove the variation introduced by BMI, the partial RDA showed that gender, age, and smoking, and metformin, antidepressant and PPI use did not significantly contribute to the separation of the fecal samples (variance explained = 21.2%, *p* = 0.31).

### 3.7. Effect of Gastric Sleeve Procedure on the Fecal Microbiota in Morbidly Obese Individuals

**Figure 6:**
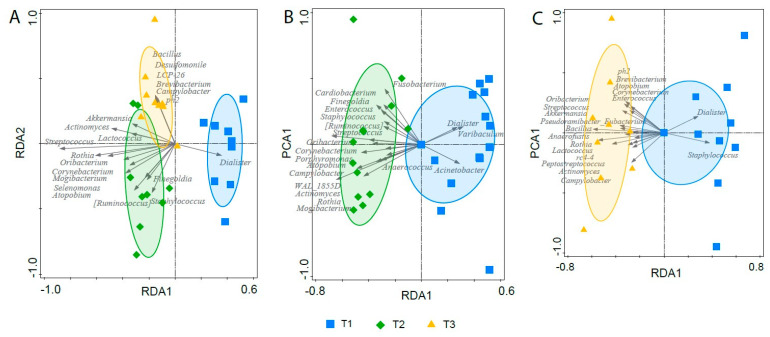
RDA based on the fecal microbiota composition of individuals pre-GS (squares), and two (diamonds) and six months (crosses) post-GS (**A**); RDA based on the fecal microbiota composition of individuals pre-GS (squares) and two months post-SG (diamonds) (**B**); and RDA based on the fecal microbiota composition of individuals pre-SG (squares) and six months post-SG (crosses) (**C**). The 20 bacterial taxa that contributed most to the separation are represented by arrows. The ellipses are 2D-normal-based ellipses at the 66% confidence level.

To further investigate the fecal microbiota change within the obese individuals post-SG, a partial RDA was conducted, in which the variable “subject” was included as a covariate to remove variation introduced by host specificity. Partial RDA on the fecal samples of obese individuals pre- and post-SG indicated that the fecal microbiota changed significantly following SG (variance explained: 19.6%, *p* = 0.008) Figure 6A). The separation was mainly driven by a decreased abundance of the genus *Dialister* and an increased abundance of *Streptococcus*, *Actinomyces* and *Rothia* in post- as compared to pre-SG (Figure S2). A list of the 20 most important taxa for the separation and their corresponding importance score can be found in Table S2.

To further elucidate the shifts in specific bacterial taxa in obese individuals following SG, the 20 top-classifying bacterial taxa from the partial RDA were selected and the changes in the relative abundance of these taxa were analyzed with the Wilcoxon signed-rank test. Of those signature genera, *Streptococcus*, *Actinomyces*, and *Rothia* were significantly increased both at two (fold increase of 14.6; *p* = 1.8 × 10^−3^, 4.6; *p* = 3.0 × 10^−3^ and 24.5; *p* = 3.7 × 10^−3^, respectively) and six months (fold increase of 11.7; *p* = 7.7 × 10^−3^, 2.8; *p* = 7.7 × 10^−3^ and 7.0; *p* = 7.7 × 10^−3^, respectively) post-SG. A significant decrease in *Dialister* was also observed at both two (fold decrease of 0.17; *p* = 1.6 × 10^−2^) and six months (fold decrease of 0.3; *p* = 7.7 × 10^−3^) post-SG. For *Corynebacterium* a weak significant increase was also observed at two (fold increase of 6.2; *p* = 0.02) and six months (fold increase of 4.2; *p* = 0.04) post-SG. *Oribacterium* was not present pre-SG, but showed a significant slight increase at two (relative abundance: 8.2 × 10^−5^, *p* = 0.02) and six months (relative abundance: 6.6 × 10^−5^, *p* = 0.04) post-SG. However, none of the changes in the relative abundance of the above-mentioned signature taxa remained statistically significant after correction for multiple testing.

To disentangle the short- and long-term effects of SG on the microbiota composition, we performed separate partial RDAs comparing the microbiota composition between: (i). pre- and two months post-SG; (ii) pre- and six months post-SG, and; (iii) two and six months post-SG, respectively.

The short-term changes in the fecal microbiota composition, as indicated by the partial RDA on pre-SG and two months post-SG samples (*p* = 0.004, Figure 6B) were mainly characterized by an increase in the relative abundance of *Streptococcus*, *Rothia*, *Actinomyces*, *Atopobium*, *Mogibacterium*, *Oribacterium*, *Carynebacterium* and *Finegoldia*, and a decrease in *Dialister*. The RDA on the pre- and six months post-SG samples demonstrated that the fecal microbiota composition was still significantly altered six months after surgery (*p* = 0.008, Figure 6C) and that these long-term alterations were still mainly driven by an increase in the relative abundances of *Streptococcus*, *Rothia* and *Actinomyces*. Other drivers for this separation were *Staphylococcus* (decreased in samples six months post-GS), *Akkermansia* and *Oribacterium* (both increased in six months post-SG samples, Figure S2). A list of the most important taxa for the separation and their corresponding importance score can be found in Tables S3 and S4.

To detect which alterations might be related to the surgery itself or to the physiological changes induced by the gastric sleeve, we compared the behavior of the top 30 taxa from the RDA comparisons of T1 vs. T2 versus T1 vs. T3 (Figure S3). These comparisons indicated that the relative abundance of *Staphylococcus* was increased two months post-SG, but decreased six months post-SG as compared to baseline (Figure S2), suggesting that *Staphylococcus* is more likely to be temporarily affected by the surgical procedure, rather than the physiological changes due to the gastric sleeve. No other bacterial taxa behaved differently two months or six months post-SG. Moreover, in agreement with the observation that the most prominent shifts in bacterial taxa following SG were observed both two and six months after surgery, partial RDA did also not demonstrate separation of the samples collected two and six months post-SG (*p* = 0.47).

At both two (*p* = 1.51 × 10^−7^) and six (*p* = 8.00 × 10^−6^) months post-SG, the F/B ratios of the obese individuals remained significantly higher as compared to the lean individuals (Figure S1). When examining the microbiota within the group of obese individuals over time, changes in the F/B ratio could neither be observed at two nor at six months post-SG as compared to baseline (Figure S1, *p* > 5.0 × 10^−2^).

### 3.8. Potential Confounding Effects on the Microbiota

Neither within the lean individuals nor within the morbidly obese individuals at baseline, were fecal protein concentration, diabetes, gender, smoking, alcohol and medication use associated with the microbiota composition, indicating that potential confounding by these variables within our study was not likely (Table S5).

### 3.9. Associations between Fecal Microbiota Composition and Biomarkers

A significant correlation was found between hsCRP and the overall fecal microbiota composition in obese individuals prior to the SG procedure (*p* = 0.002). Such correlation was not found in the lean (*p* = 0.632) nor in the in obese individuals two (*p* = 0.27) and six (*p* = 0.26) months post-SG. For fecal calprotectin, no such correlations were found. Furthermore, no correlation was found between fecal calprotectin, Hba1c or intestinal permeability markers and the microbiota composition of all groups (Table S6).

Despite the absence of an association with the overall microbiota composition, a Pearson product-moment correlation analysis was performed in the morbidly obese group, to examine whether changes in glycemic control (i.e., plasma Hba1c), inflammatory (plasma hsCRP and fecal calprotectin) and intestinal permeability markers were associated with the microbial taxa that were most strongly affected by the SG (i.e., *Streptococcus*, *Rothia* and *Actinomyces*). The relative change in abundance of *Streptococcus* between baseline and two months post-SG was positively correlated with Hba1c, but did not remain after correction for multiple testing (*p* = 0.01, FDR corrected 0.17). No further correlations were found between the bacterial taxa and permeability nor with inflammatory markers (Table S7).

## 4. Discussion

We have compared the fecal microbiota between morbidly obese and lean individuals and subsequently investigated the impact of sleeve gastrectomy on fecal microbiota and on biomarkers over time. Our results confirm that morbidly obese individuals have a microbiota composition that is distinct from lean individuals. Furthermore, their microbiota composition changed significantly following SG and these alterations persisted up to six months post-surgery. However, the post-SG microbiota did not change towards a lean profile, nor did the microbial alterations post-SG correlate with markers of inflammation, glycemic control or intestinal permeability.

Compared to lean individuals, the microbiota of morbidly obese individuals prior to SG was characterized by a reduced microbial diversity, a lower abundance of *Bacteroides*, *Sutterella*, *Lachnospira* and *Butyricimonas* and a higher F/B ratio, which is in agreement with findings of several previous studies [9,43,44,45,46]. Moreover, a higher relative abundance of several SCFA-producing genera, including *Eubacterium*, *Dorea*, *Ruminococcus*, and *Blautia*, was observed in morbidly obese as compared to lean individuals. Higher fecal SCFA levels have been reported in obese individuals, being supportive for increased energy harvest as a potential contributive factor [6,12,47,48,49]. BMI was found to be driving the separation of the microbiota between morbidly obese and lean individuals, whereas gender, age, smoking and medication use as confounding factors did not contribute.

Overall, the fecal microbiota structure at two and six months post-SG changed profoundly as compared to pre-SG, but not towards the microbial profiles of lean subjects. Previous studies have shown that alterations in the microbiota of obese subjects following SG and RYGB are also not towards a lean, healthy microbiota composition [9,46]. Dietary restriction, however, has been reported to change the microbiota of obese subjects towards a lean microbiota profile [10,50]. Unfortunately, detailed dietary intake data were not available for our population. We did check for other potential confounders, but the microbiota profiles pre- and post-SG did not correlate with fecal protein concentrations as marker of fecal consistency, gender, alcohol use, smoking and medication use, including metformin, a diabetic medication previously shown to have an effect on gut microbiota [51]. Due to a low number of participants using metformin, possible confounding effects of metformin cannot be ruled out.

Although some bacterial taxa were only altered at either two or six months post-SG, the majority of the microbial changes were consistent at two and six months post-SG, supporting the notion that the microbiota changes are not merely a transient effect of the surgical procedure or antibiotic prophylaxis, but more likely result from a combination of changes in gastrointestinal physiology, function and the diet. An increase in facultative anaerobic bacteria characteristic for the upper gastrointestinal tract, including *Streptococcus*, *Rothia* and *Actinomyces*, were amongst the most discriminative features of the microbiota post as compared to pre-SG. Similar findings of an increase in facultative anaerobic bacteria were also noted by others, after SG [52,53] and also after RYGB (mainly Proteobacteria) [28,29,30,31,54,55].

Up to now, several studies have reported on fecal microbiota profiles after SG. In general, the microbial changes appear to be less pronounced after SG compared to RYGB. Increased species diversity has been reported [3,4,5,11,12,16], while others found no changes in diversity [14,15,19,27]. With respect to microbial composition, *Akkermansia* [2,5,33] *Bacteroidetes* [2,3,7,12,20], and *Clostridium* [2,6] were found to be increased while Firmicutes [2,14] and Prevonella [2]) were found to be decreased. The increase in facultative anaerobic bacteria may result from a decreased gastric acid production and accelerated gastric transit time reported after the SG procedure, which could promote the survival of upper GI microorganisms, resulting in an increase in these GI microorganisms in the colon [56,57]. Damms-Machado et al. [58] observed a reduction in butyrate-producing bacteria in several obese individuals post-SG, whereas these bacterial taxa were not found to be significantly affected in our study. These apparently discordant findings might (in part) be explained by the differences in dietary intake. Participants in the study by Damms-Machado et al. followed a strict postoperative nutritional care program, including a diet low in fiber and starch, which could explain the reduction in SCFA producers [58].

The gastric sleeve procedure is thought to leave the intestinal tract distal from the pylorus intact [59]; therefore we hypothesized that changes in the microbiota, in contrast to microbiota alterations upon RYGB, are more likely associated with weight loss and less impacted by changes in anatomy. Recently, we showed that after SG the small intestinal transit time becomes significantly accelerated, providing additional new information on the effect of SG on GI physiology (Wilbrink et al., unpublished data).

Gastroduodenal permeability, measured by urinary sucrose recovery, was increased in obese individuals versus controls [35]. After surgery urinary sucrose recovery decreased to values not different from controls. Whether this reflects a true amelioration in gastroduodenal permeability or is the result of an accelerated gastrointestinal transit cannot be concluded from our findings. In contrast to our findings, Verdam et al. [59] did not find a relation between BMI and sucrose permeability. When analyzing their study in more detail, the gastroduodenal permeability was twice as high in the obese compared to the non-obese group [60].

In our population of morbidly obese subjects, small bowel and colonic permeability was not affected. These findings are in line with previously published data from other groups comparing lean and obese individuals [30,60,61,62,63,64]. The urinary lactulose/rhamnose ratio decreased significantly at 6 months after SG compared to preoperative values, but whether this reflects an improvement in permeability or is the result of an acceleration of intestinal transit time after SG cannot be concluded based on our data. No correlations were found between changes in permeability and gut microbiota composition.

A correlation between the obese microbiota prior to SG with hsCRP was found, suggesting a relation specifically between the obesogenic microbiota composition and systemic inflammation. However, the changes in biomarkers for systemic and intestinal inflammation, intestinal permeability and glycemic control following SG did not correlate with shifts in the microbiota composition. Our findings are in line with those of Kellerer et al., who found no link between microbiota composition and gut permeability post-SG [30], whereas others have reported correlations between specific bacterial taxa and metabolic or inflammatory markers [11,29,32,33]. Despite significant weight loss and improvements in inflammatory and metabolic markers in the present study, the microbiota composition did not change towards a lean microbiota profile.

Here, we have shown that an SG procedure, via subsequent changes in gastrointestinal physiology and function, significantly affects the fecal microbiota composition of morbidly obese individuals. These changes include a decreased production of gastric acid and an increased gastrointestinal transit time, which could promote the survival of the oral microbiota [65,66]. Also, an altered bile acid composition has been reported after sleeve gastrectomy [67]. The composition of gut microbiota can be influenced by bile acid composition and vice versa [68,69]. Therefore, we cannot exclude the possibility that the gut microbiome changes also impact metabolism and thereby host health. This includes, e.g., impact on acid secretion, bile acid metabolism, and glucose and insulin metabolism [15,29]. To what extent these microbial shifts might impact host health needs to be investigated in future mechanistic studies. Although individuals in the operative group showed a significant weight loss, the median BMI six months post-SG (i.e., 34.8) still points towards an obese phenotype, and a more prolonged follow-up period is necessary to investigate whether the microbiota changes induced by the SG procedure are persistent even after a BMI has been achieved within or close to the physiological ranges.

Several strengths of our study should be mentioned. First, the longitudinal design enabled us to monitor the microbial changes upon sleeve gastrectomy within the same individuals over time. Second, by including an age- and gender-matched lean control group, we were able to compare baseline and SG-induced microbial alterations with the microbiota composition in lean individuals. Third, we included clinical parameters and biomarkers for inflammation, metabolism and the intestinal barrier. Therefore, we were able to control for the effect of known potential confounders, such as diabetes, medication use, gender and smoking [70]. A limitation of our study is the lack of detailed information on dietary intake before and after the gastric sleeve procedure. Therefore, we cannot exclude that the observed microbial changes were (partially) affected by altered dietary intake. The detailed assessment of dietary intake is essential in future studies investigating the effect of bariatric surgery on the microbiota, considering the significant impact diet has on the microbiota composition and activity [71].

In conclusion, we found a significant shift in fecal microbiota composition of individuals with morbid obesity following SG, but not towards a lean fecal microbiota profile. The post-SG fecal microbiota profile was characterized by an increase in facultative anaerobic bacteria, characteristic for the upper gastrointestinal tract. This change might be explained by the functional changes induced by the SG procedure. No correlations were found between changes in microbial composition and metabolic, inflammatory or intestinal-barrier biomarkers.

## Figures and Tables

**Table 1:** Clinical characteristics of the study population.

	Lean Subjects (N = 27)	Subjects with Obesity T1 (N = 24) pre-GS	T2 (N = 13) Two Months Post-GS	T3 (N = 9) Six Months Post-GS
BMI (kg/m^2^) (median, IQR)	23.0 (21.5–24.4) *	43.5 (39.4–48.3) *$	37.9 (36.0–43.0) *$	34.8 (31.5–39.4) *$
Total weight loss (%) (median, IQR)	Not applicable	Not applicable	14.8 (11.8–20.4)	25.1 (21.4–27.4)
Age (yrs) (median, IQR)	47 (24–61)	43 (37–55)	50 (41.5–55)	50.0 (42–55)
Male, number (%)	12 (44.4)	11 (45.8)	7 (53.8)	5 (55.6)
Current smokers, n (%)	3 (11.1)	7 (29.2)	3 (23.1)	3 (33.3)
Hypertension n (%)	0 (0) #	14 (58.3) #	9 (69.2) #	5 (55.6) #
Type 2 diabetes (n, %)	0 (0) #	10 (41.7) #	6 (46.2) #	4 (44.4) #
Medication (n, %)				
Insulin	0 (0) #	4 (16.7)#	3 (23.1)	0 (0)
Oral antidiabetics ^1^	0 (0)	13 (54.1)	7 (53.8)	8 (88.9)
PPI use (%) ^2^	1 (3.7)	2 (3.8)	3 (23.1)	1 (11.1)
Antibiotics ^3^	0 (0)	0 (0)	0 (0)	0 (0)
Past antibiotics ^4^	1 (3.7) #	0 (0)	0 (0)	1 (11.1)
Plasma markers				
hsCRP (mg/l, median, IQR)	0.6 (0.4–0.9) *	6.3 (3.0–4.0) *$	7.3 (5.4–11.2) *&	3.4 (1.9–4.1) *$&
Hba1c (mM Hba1c/M HB median, IQR)	Not available	49 (40–73) $	41 (35–55) $	43 (33–53) $
Fecal markers				
Calprotectin (µg/gram feces, median, IQR)	1.4 (0.8–3.4) *	3.7 (2.0–9.7) *$&	15.6 (11.9–21.0) *$	10.7 (8.6–13.7) *&
Protein content (µg protein/mg feces, median, IQR)	117.4 (92.2–192.5)	127.0 (68.5–200.6)	162.6 (52.7–237.6)	162.2 (101.2–231.4)
Urinary markers				
Sucrose (µg, median, IQR)	1958.8 (1042.8–3716.6) *	5642.1 (1956.3–24,273.7) *	1846.2 (450.7–4565.1)	2758.5 (1174.0–5694.4)
Lactulose/rhamnose (median, IQR)	0.04 (0.03–0.08)	0.05 (0.02–0.07) $	0.05 (0.03–0.06) &	0.045 (0.04–0.10) $&
Sucralose/erythritol (median, IQR)	0.06 (0.02–0.08)	0.05 (0.02–0.06) $	0.08 (0.03–0.09) $	0.04 (0.02–0.06)

^1^ Oral medication (metformin, sulphonylurea derivates and thiazolidinedione. ^2^ Stopped 1 week prior to fecal sampling. ^3^ Antibiotic use at moment of sampling. ^4^ Antibiotics use between 8 and 4 weeks prior to sampling moment. * *p* < 0.05 according to Mann–Whitney U test HC vs. T1; HC vs. T2; HC vs. T3. # *p* < 0.05 according to chi squared test between HC vs. T1; HC vs. T2; HC vs. T3. $ and & *p* < 0.05 according to Wilcoxon signed-rank test. Identical symbol indicates significant differences between 2 groups.

## Data Availability

In line with the ethics approval for the current study, data can be made available upon a reasonable request. Deidentified data can then be shared upon a Data Transfer Agreement and as long as analyses are in line with the ethics permission. Requests to access these datasets should be directed to the corresponding author (a.masclee@mumc.nl).

## Supplementary Materials

The following supporting information can be downloaded at: https://www.mdpi.com/article/10.3390/microorganisms11092353/s1, Figure S1. The fecal Firmicutes/Bacteroidetes (F/B) ratio in healthy individuals (HC), individuals pre-SG (T1), individuals post-SG two months (T2) and six months (T3) post-GS. Data are presented as boxplots displaying medians with interquartile ranges. ** indicates *p* < 0.0001; Figure S2. Relative abundances of specific taxa at specified time points (pre-SG (T1), two (T2) and six (T3) months post-SG); Figure S3. Scatterplot based on the coordinates of the most important taxa according to the RDAs comparing samples T1 (pre-SG) versus T2 (2 months post-SG) and RDAs comparing samples T1 (pre-SG) versus T3 (6 months post-SG); Table S1. Taxa that contributed most to the separation between healthy individuals (HC) and obese individuals pre-SG (T1) according to their importance scores; Table S2. Taxa that contributed most to the separation between obese individuals pre-SG (T1), two months (T2) and six months (T3) post-SG according to their importance scores; Table S3. Taxa that contributed most to the separation between obese individuals pre-SG (T1) and obese individuals two months (T2) post-SG; Table S4. Taxa that contributed most to the separation between obese individuals pre-SG (T1) and obese individuals six months (T3) post-SG; Table S5. Confounder analysis of patient characteristics by means of RDA; Table S6. Associations between host markers and the microbiota composition as examined by CCA; Table S7. Pearson correlation coefficients (R) between changes in the three most strongly affected taxa by SG and markers of glycemic control, inflammatory and intestinal permeability.
