# Supplementary material for: Impact of Sleeve Gastrectomy on Fecal Microbiota in Individuals with Morbid Obesity
Source: Microorganisms. 2023 Sep 20;11(9):2353. doi: 10.3390/microorganisms11092353 (PMC10537490; doi:10.3390/microorganisms11092353)
Supplement: Supplementary file 1 [file microorganisms-11-02353-s001.zip › microorganisms-2501284-supplementary.pdf]

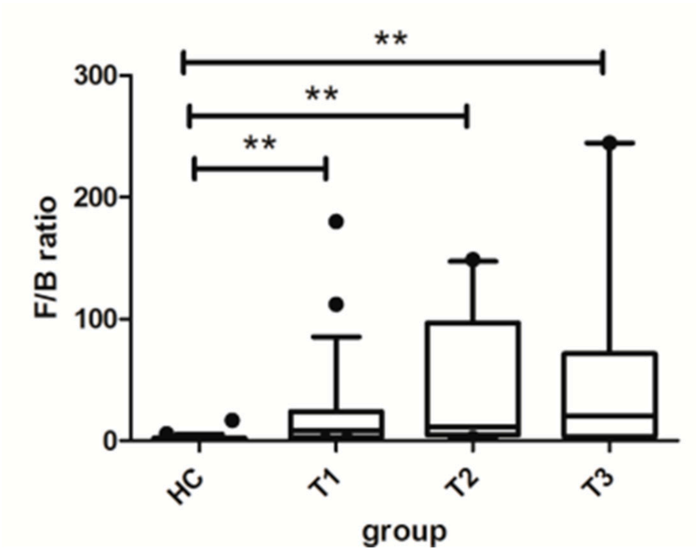


**Supplemental Figure S1**. The fecal Firmicutes/Bacteroidetes (F/B) ratio in healthy individuals (HC), individuals pre-SG (T1), individuals post-SG two months (T2) and six months (T3) post-GS. Data are presented as boxplots displaying medians with interquartile ranges. ** indicates p<0.0001

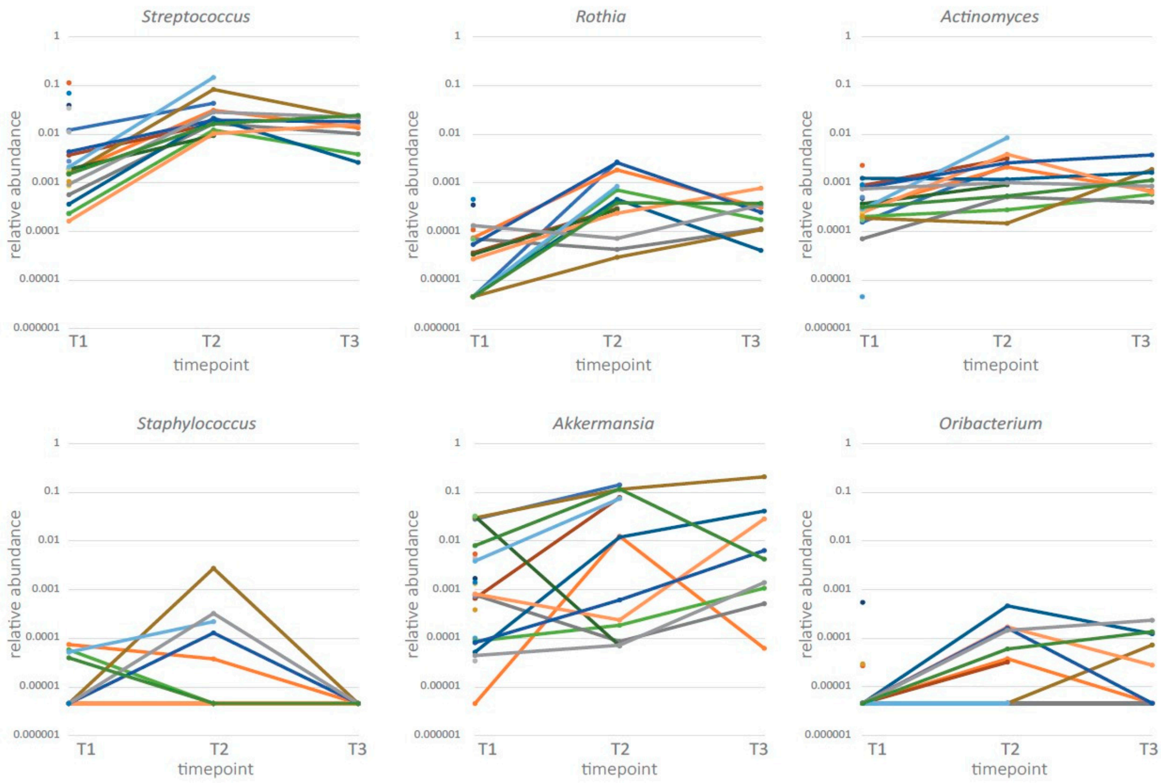


**Supplemental Figure S2.** Relative abundances of specific taxa at specified time points (pre-SG (T1), two (T2) and six (T3) months post-SG). Horizontal axis: “timepoint”, vertical axis: “relative abundance”.

versus T3 (6 months post-SG).

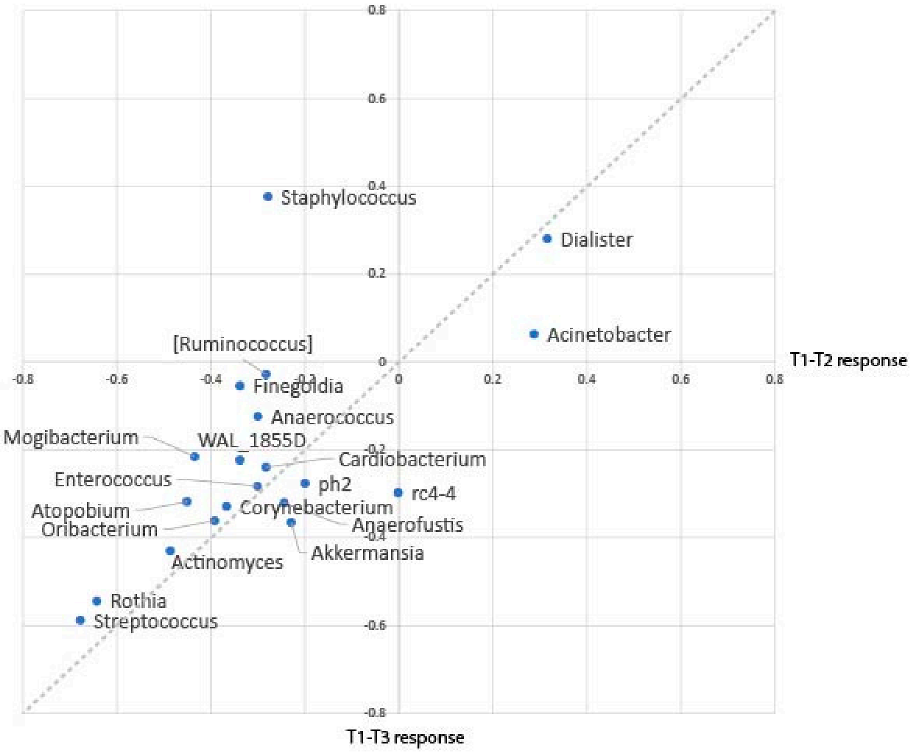


**Supplemental Figure S3.** Scatterplot based on the coordinates of the most important taxa according to the RDAs comparing samples T1 (pre-SG) versus T2 (2 months post-SG) and RDAs comparing samples T1 (pre-SG)

**Supplemental Table S1. Taxa that contributed most to the separation between healthy individuals (HC) and obese individuals pre-SG (T1) according to their importance scores.**

| Taxa important for HC vs T1 | importance score |
|---|---|
| **Odoribacter** | 0.7567 |
| **Bacteroides** | 0.7068 |
| **[Eubacterium]** | 0.6681 |
| **Sutterella** | 0.6581 |
| **Lachnospira** | 0.6467 |
| **Butyricimonas** | 0.6310 |
| **Actinomyces** | 0.6228 |
| **Dorea** | 0.6014 |
| **[Ruminococcus]** | 0.5914 |
| **Parabacteroides** | 0.5664 |
| **Blautia** | 0.5059 |
| **Peptococcus** | 0.3183 |
| **Anaerostipes** | 0.3045 |
| **Paraprevotella** | 0.2640 |
| **Bulleidia** | 0.2634 |
| **Mitsuokella** | 0.2115 |
| **Catenibacterium** | 0.1597 |
| **Desulfovibrio** | 0.1356 |

| | |
|---|---|
| **[Prevotella]** | 0.0412 |
| **Prevotella** | 0.0235 |

**Supplemental Table S2. Taxa that contributed most to the separation between obese individuals pre-SG (T1), two months (T2) and six months (T3) post-SG according to their importance scores.**

| Taxa important for T1 vs T2 vs T3 | importance score |
|---|---|
| **Streptococcus** | 0.8789 |
| **Rothia** | 0.6062 |
| **Oribacterium** | 0.5109 |
| **Actinomyces** | 0.4825 |
| **Corynebacterium** | 0.3900 |
| **Atopobium** | 0.3757 |
| **Dialister** | 0.3535 |
| **Mogibacterium** | 0.3439 |
| **Lactococcus** | 0.3305 |
| **Akkermansia** | 0.3239 |
| **[Ruminococcus]** | 0.3101 |
| **Finegoldia** | 0.2277 |
| **Selenomonas** | 0.2121 |
| **Staphylococcus** | 0.1754 |
| **Brevibacterium** | 0.1513 |
| **Campylobacter** | 0.1500 |
| **Bacillus** | 0.1467 |
| **Desulfomonile** | 0.1448 |
| **ph2** | 0.1426 |
| **LCP-26** | 0.1387 |

**Supplemental Table S3. Taxa that contributed most to the separation between obese individuals pre-SG (T1) and obese individuals two months (T2) post-SG.**

| Taxa important for T1 vs T2 | importance score |
|---|---|
| **Streptococcus** | 0.6780 |
| **Rothia** | 0.6427 |
| **Actinomyces** | 0.4866 |
| **Atopobium** | 0.4513 |
| **Mogibacterium** | 0.4347 |
| **Oribacterium** | 0.3923 |
| **Corynebacterium** | 0.3667 |
| **WAL_1855D** | 0.3384 |
| **Finegoldia** | 0.3382 |
| **Dialister** | 0.3156 |
| **Enterococcus** | 0.3013 |
| **Anaerococcus** | 0.3004 |
| **Acinetobacter** | 0.2875 |
| **[Ruminococcus]** | 0.2830 |
| **Cardiobacterium** | 0.2828 |

| | |
|---|---|
| **Staphylococcus** | 0.2786 |
| **Fusobacterium** | 0.2750 |
| **Varibaculum** | 0.2697 |
| **Campylobacter** | 0.2670 |
| **Porphyromonas** | 0.2604 |

**Supplemental Table S4. Taxa that contributed most to the separation between obese individuals pre-SG (T1) and obese individuals six months (T3) post-SG.**

| Taxa important for T1 vs T3 | importance score |
|---|---|
| **Streptococcus** | 0.5878 |
| **Rothia** | 0.5444 |
| **Actinomyces** | 0.4298 |
| **Staphylococcus** | 0.3768 |
| **Akkermansia** | 0.3652 |
| **Oribacterium** | 0.3610 |
| **Brevibacterium** | 0.3434 |
| **Corynebacterium** | 0.3286 |
| **Anaerofustis** | 0.3205 |
| **Atopobium** | 0.3178 |
| **rc4-4** | 0.2976 |
| **Enterococcus** | 0.2828 |
| **Dialister** | 0.2811 |
| **ph2** | 0.2760 |
| **Campylobacter** | 0,2683 |
| **Peptostreptococcus** | 0.2594 |
| **Lactococcus** | 0.2571 |
| **Bacillus** | 0.2554 |
| **Pseudoramibacter_Eubacterium** | 0.2412 |

**Supplemental Table S5. Confounder analysis of patient characteristics by means of RDA.**

| Variable tested | Study group | Explained variation (%) | P-value |
|---|---|---|---|
| **Diabetes** | T1 | 3.2 | 0.84 |
| **Metformin** | T1 | 5.4 | 0.20 |
| **Metformin** | T2 | 8.3 | 0.46 |
| **Metformin** | T3 | 15.4 | 0.19 |
| **Gender** | HC | 2.9 | 0.78 |
| **Gender** | T1 | 4.0 | 0.52 |
| **Smoking** | T1 | 8.3 | 0.57 |
| **Antidepressants** | T1 | 6.0 | 0.98 |
| **PPI** | T1 | 4.1 | 0.54 |

**Supplemental Table S6. Associations between host markers and the microbiota composition as examined by CCA.**

| Variable tested | Study group | Explained variation (%) | P-value |
|---|---|---|---|
| **Calprotectin** | HC | 6.2 | 0.05 |
| **Calprotectin** | T1 | 4.8 | 0.29 |
| **Calprotectin** | T2 | 6.9 | 0.84 |
| **Calprotectin** | T3 | 15.0 | 0.17 |
| **hsCRP** | HC | 3.0 | 0.63 |
| **hsCRP** | T1 | 9.7 | 0.002 |
| **hsCRP** | T2 | 9.2 | 0.27 |
| **hsCRP** | T3 | 13.9 | 0.26 |
| **L/R ratio** | HC | 3.6 | 0.56 |
| **L/R ratio** | T1 | 4.2 | 0.47 |
| **L/R ratio** | T2 | 9.4 | 0.40 |
| **L/R ratio** | T3 | 15.5 | 0.10 |
| **protein content** | HC | 4.2 | 0.31 |
| **protein content** | T1 | 4.6 | 0.37 |
| **protein content** | T2 | 11.2 | 0.24 |
| **protein content** | T3 | 14.7 | 0.26 |
| **Hba1c** | T1 | 4.9 | 0.62 |
| **Hba1c** | T2 | 11.7 | 0.22 |
| **Hba1c** | T3 | 18.0 | 0.07 |
| **S/E ratio** | HC | 1.4 | 0.31 |
| **S/E ratio** | T1 | 3.3 | 0.89 |
| **S/E ratio** | T2 | 6.2 | 0.92 |
| **S/E ratio** | T3 | 13.1 | 0.36 |
| **sucrose** | HC | 3.7 | 0.51 |
| **sucrose** | T1 | 4.5 | 0.48 |
| **sucrose** | T2 | 9.9 | 0.17 |
| **sucrose** | T3 | 11.5 | 0.68 |

**Supplemental Table S7. Pearson correlation coefficients (R) between changes in the three most strongly affected taxa by SG and markers of glycemic control, inflammatory and intestinal permeability.**

| | Streptococcus | | | Actinomytes | | | Rothia | | |
|---|---|---|---|---|---|---|---|---|---|
| Variable | R | p | FDR | R | p | FDR | R | p | FDR |
| **hba1c ΔT1-ΔT2** | 0.796 | 0.010 | 0.225 | -0.154 | 0.693 | 0.846 | -0.560 | 0.117 | 0.642 |
| **hba1c ΔT1-ΔT3** | 0.526 | 0.180 | 0.692 | -0.520 | 0.186 | 0.692 | 0.307 | 0.460 | 0.764 |
| **CRP ΔT1-ΔT2** | 0,314 | 0.300 | 0.705 | 0.021 | 0.946 | 0.968 | -0.152 | 0.620 | 0.801 |
| **CRPc ΔT1-ΔT3** | 0.384 | 0.308 | 0.713 | -0.532 | 0.141 | 0.687 | 0.474 | 0.198 | 0.692 |
| **calprotectin ΔT1-ΔT2** | 0.176 | 0.565 | 0.801 | 0.240 | 0.429 | 0.738 | 0.409 | 0.166 | 0.692 |
| **calprotectin ΔT1-ΔT3** | 0.417 | 0.264 | 0.692 | -0.429 | 0.249 | 0.692 | 0.654 | 0.056 | 0.434 |
| **sucrose ΔT1-ΔT2** | 0.158 | 0.625 | 0.720 | -0.343 | 0.276 | 0.434 | -0.584 | 0.046 | 0.738 |
| **sucrose ΔT1-ΔT3** | 0.629 | 0.070 | 0.801 | -0.669 | 0.049 | 0.692 | 0.303 | 0.428 | 0.434 |
| **SE ΔT1-ΔT2** | 0.183 | 0.549 | 0.801 | 0.091 | 0.767 | 0.855 | -0.112 | 0.717 | 0.852 |

| | | | | | | | | | |
|---|---|---|---|---|---|---|---|---|---|
| **SE ΔT1-ΔT3** | 0.007 | 0.986 | 0.986 | 0.298 | 0.436 | 0.738 | -0.109 | 0.779 | 0.855 |
| **LR ΔT1-ΔT2** | 0.285 | 0.369 | 0.738 | 0.354 | 0.260 | 0.692 | 0.161 | 0.617 | 0,801 |
| **LR ΔT1-ΔT3** | -0.318 | 0.405 | 0.738 | 0.236 | 0.541 | 0.801 | -0.112 | 0.774 | 0.855 |
| **BMI ΔT1-ΔT2** | 0.376 | 0.229 | 0.801 | 0.161 | 0.617 | 0.924 | -0.297 | 0.349 | 0.801 |
| **BMI ΔT1-ΔT3** | 0.214 | 0.580 | 0.692 | 0.069 | 0.861 | 0.801 | -0.220 | 0.569 | 0.732 |
